# Examining the relationship between empathy and burnout in dental students: a systematic review

**DOI:** 10.1038/s41432-025-01182-z

**Published:** 2025-08-08

**Authors:** Sviatlana Anishchuk, Aidan Seery

**Affiliations:** 1 Trinity College Dublin, The University of Dublin, Ireland; Dublin Dental University Hospital, Dublin, Ireland; 2 Trinity College Dublin, The University of Dublin, Dublin, Ireland

**Keywords:** Dental education, Dentistry, Health care

## Abstract

**Objectives:**

Empathy has an important role in the patient-clinician relationship. However, there is a risk of an emotional overabundance, which can lead to a burnout and exhaustion. The nature of its relationship is yet not clear. This study aims to investigate the relationship between empathy and burnout in dental undergraduate students.

**Methods:**

A systematic review was carried out in accordance with Preferred Reporting Items for Systemic Reviews and Meta-Analyses (PRISMA) using electronic searches on electronic databases: PubMed (PMC), Taylor and Francis, Google Scholar and EBSCO. The databases were examined for studies that were published in English language from 2012 to 2022.

**Results:**

There were 2375 articles in total. The studies that only assessed empathy or burnout and targeted other than dental cohorts were excluded from the list. Any studies that were not in English language and had been published before 2012 were also excluded. As a result, only 2 studies were selected for further analysis.

**Conclusion:**

Based on this review it can be assumed that the level of burnout depends on the level of empathy in dental cohort students; low empathy can predict high burnout. However, further research is necessary to confirm this relationship.

Key Points

- The relationship between empathy and burnout is interrelated, implying that cognitive empathy can serve as a protective factor to exhaustion and burnout in dental healthcare profession.
- The review suggested that empathy can predict a burnout in the dental undergraduates.
- An appropriate educational intervention could help to enhance cognitive empathy in dental undergraduate training therefore to prevent early professional burnout.

## Introduction

Empathy is an important competency in the healthcare profession and one of the main elements of effective patient-clinician relationship^1,2^. Although the term has been widely discussed in the literature, however, to date, there is no unity in the definition of empathy particularly in patient care^3^. Empathy can be described as being able to understand and share the emotions of another person without actually experiencing them. While sympathy, on the other hand, is the process of feeling sorrow for someone else’s experience^4^ that can lead to an overabundance that may result in a clinician’s compassion fatigue, exhaustion (burnout), and vicarious trauma^5,6^. In terms of patient care, empathy is defined as a cognitive attribute that implicates an understanding of the patient’s suffering, and ability to communicate back this understanding with the further intention to help. While sympathy is different as it entails feeling of the patient’s suffering^7,8^. Due to an emotional nature of sympathy, there is a risk of an overabundance, which can be overwhelming and for an example, may hinder the clinician’s performance^7^ and lead to emotional exhaustion, depersonalisation and burnout^9^. The level of burnout was reported to be high in dental science students’ cohort^10^. This is concerning, as it can have an impact on their personal and psychological health^11^ as well as also affect the clinician-patient relationship which could lead to a medical error, low empathy and patient care^12^. This is not surprising, as emotional exhaustion is one of the domains of the burnout phenomenon. The inverse correlations between empathy and depersonalisation scores of the MBI were reported previously^13–15^.

There are numerous research tools to measure empathy and burnout. One of the most widely tools that are used to assess empathy is the interpersonal reactivity index (IRI)^16^ that covers perspective-taking, empathic concern, personal distress and fantasy. There are other scales, for example empathy scale from Hogan^17^, emotional empathy scale^18^, balanced emotional empathy scale (BEES)^19^ and more. Some tools include empathy as a subscale, one of them is an emotional quotient self-assessment checklist (EQSAC)^20^. However, most of these scales are aimed either to assess an empathetic level of a general population or are part of the bigger scales and predominately designed to assess an emotional empathy or not indicative on the type of empathy, although IRI assess both elements, cognitive and emotional empathy. However, the Jefferson scale of empathy (JSE) was specifically designed to assess an empathy level in health care professionals^21^.

An extensive work of Jackson and Maslach led to developing an inventory for measuring hypothesised aspects of burnout in humans, the Maslach burnout inventory (MBI)^22^, then later became modified and named as MBI-Human Services Survey (MBI-HSS) and reduced to 22 items: EE 9 items, DP 5 items and PA 8 items. Further version of MBI was modified to use in educational services and named MBI-Educators Survey (MBI-ES)^23^. Both were designed to focus on assessing burnout in an occupation that involved interaction with people for instance for students in educational settings or healthcare professionals dealing with patients. The Copenhagen burnout inventory (CBI)^24^ contains the assessment of three types of burnout: personal, work and client, with 19 items in total. The scale assesses personal related burnout that refers to physical and psychological exhaustion and includes 6 items, work related burnout that derived from work with 7 items and client-related burnout that results from a relationship with the clients and covers 6 items. However, CBI inventory claimed that burnout is both professional (work and client) and personal, opposed to just a general “exhaustion” which was described in MBI. Both inventories (MBI and CBI) have been validated and used widely and translated into different languages.

## Rationale

This paper is to provide a review that aims to systematically explore the relationship of empathy and burnout in dental students. The patient, exposure, comparison and outcome (PECO) search strategy was used to identify key words and formulate the question^25^, see Table 1 for more details.

**Table 1:** PECO search items.

Population of interest	Dental students
Exposure	Empathy
Comparison	None
Outcome	Burnout

Specifically, the review considered the following questions:

- What studies exist that measure empathy and burnout in dental students?
- To what extent do these studies establish dependent relationship between level of empathy and burnout?
- Do these studies indicate that a low level of empathy can predict a burnout?

## Methods

### Information sources and Protocol

The review was organised as a set of electronic searches of five electronic databases: PMC, Taylor and Francis, Google Scholar and EBSCO. The review adhered to the Preferred Reporting Items for systematic reviews and meta-analyses (PRISMA)^26^. Additionally, the search was expanded manually by identifying eligible articles that met the following terms and criteria.

### Selection of studies

Following terms were used in electronic search:

- Empathy and burnout in dental students
- Empathy and burnout
- Empathy and burnout in dentistry
- Emotional intelligence and stress
- Emotional intelligence and burnout in dental

Inclusion criteria

- English language
- Year to start from 2012 until 2022
- Open access
- Free full text
- Clinical Trial
- Randomized Controlled Trial
- Participants: dental students

Exclusion criteria

- Books and documents
- Abstracts only
- Research articles that have been published before 2012
- Not English language

**Fig. 1:**
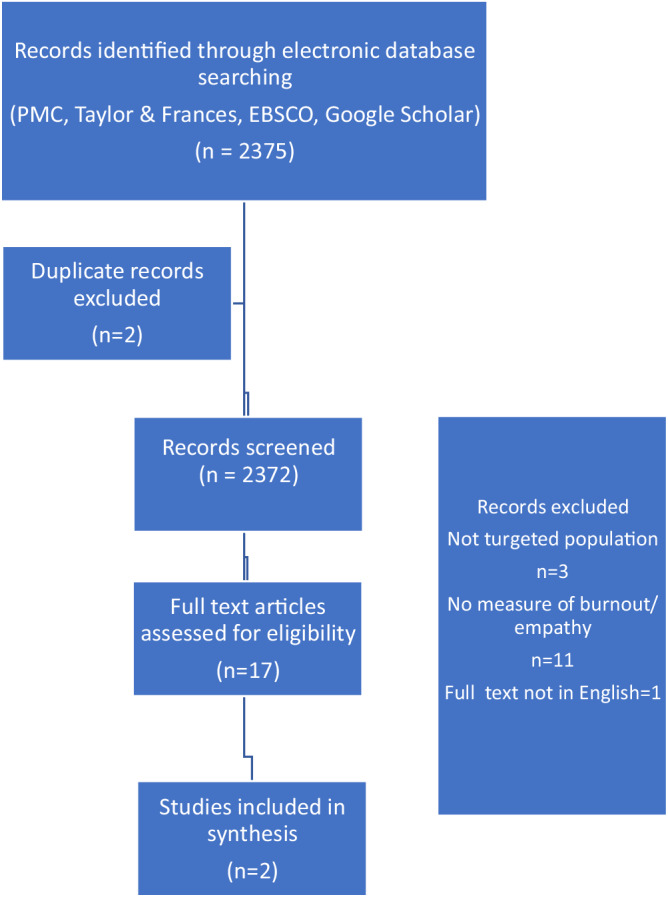
Flow chart of systematic review process.

Screened full text articles were examined and references were checked for additional articles, however none were identified. The studies that only assessed empathy or burnout were excluded from the list and studies that targeted other cohorts were excluded as well. The studies that used validated measuring tools for empathy and burnout were noted. In the research burnout and exhaustion were noted as a similar term. As a result, only two studies were selected. Prisma diagram (Fig. 1) is presented below with an explanation of the search strategy.

### Data collection process and data items

Data extraction was completed by one individual (SA). Data extracted included: title; author; year published; aim; country; sample; measuring scale; study design; profession; gender. These results were interpreted by two investigators (S.A., A.S.).

All studies were manually checked, and all identified duplicated papers were removed. Google Scholar was used to find publications that were not listed in databases. Titles and abstracts were screened to attain relevant articles for further analysis. The search included both qualitative and quantitative studies. Only studies that have dental students (undergraduate and postgraduate) were included, students from other healthcare sectors such as medical, nursing, pharmacy, paramedics etc. were excluded. Studies that were not in English and had been published before 2012 were also excluded. The studies that lacked details to determine suitability were not included in this review. These resulted in 2375 articles in total of electronic search.

## Results

Two papers were selected and evaluated, see below data extraction table, Table 2.

**Table 2:** Data extraction table.

Reference	Aim	Country	Sample	Scale	Study design	Profession	Gender
Study A-Chalikkandy et al.^27^ 10.3390/app12031588	To assess burnout and its relation to emotional dysregulation and social cognition among undergraduate dental students	Saudi Arabia	148	Copenhagen burnout inventory questionnaire (CBI), Emotion regulation questionnaire (ERQ), Interpersonal reactivity index (IRI)	Cross-sectional	Dental Science students (40 dental interns and 108 dental undergraduates)	Female
Study B-Partido and Owen^28^ Relationship between emotional intelligence, stress, and burnout among dental hygiene students— PubMed (nih.gov)	Relationship between EI, stress and burnout levels among undergraduate dental hygiene students	USA	57	Emotional Quotient Self-Assessment Checklist (EQSAC), Modified Dental Environment Stress Questionnaire (M-DESQ), Maslach Burnout Inventory-Health Services Survey (MBI-HSS)	Cross-sectional	Dental Hygiene students	Female

Both studies were cross-sectional and published between 2012–2022. Participants from study A^27^ were recruited from undergraduate course and dental intern programme, 108 dental undergraduates and 40 dental interns respectively. The forty dental interns were not included in this review. Participants in study B^28^ were all dental hygiene students, which contained 57 participants. All of the participants were females, this was either due to cultural reasons or absence of males on the course.

The empathy and burnout were measured by different scales. Both studies reported correlational analyses between burnout and empathy. Study A^27^ used Copenhagen burnout inventory questionnaire (CBI), Emotion regulation questionnaire (ERQ), Interpersonal reactivity index (IRI). Comparing burnout and emotion regulation revealed negative correlation (CBI—personal burnout *r* = −0.251; *r* = −0.220 respectively). Study B^28^ used emotional quotient self-assessment checklist (EQSAC), Modified Dental Environment Stress Questionnaire (M-DESQ), Maslach Burnout Inventory-Health Services Survey (MBI-HSS) for assessing empathy and burnout levels. In this study the correlation between empathy and emotional exhaustion (*r*(57) = −0.295, *P* < 0.05) was found to be significantly moderate.

## Discussion

This review explored the current literature on empathy and its relationship to a burnout in a dental student cohort.

The first question in the review sought to determine the existence of studies that measure empathy and burnout in dental students. There were only two studies identified that measured empathy and burnout in dental cohort and they are included in this review. The study A^27^ from a developing country (Saudi Arabia) and study B^28^ from a developed country (USA). The size of the studies is different, there were 102 in study A versus 57 in study B participants. The participants also differ in their dental speciality, study A investigated dental science students, study B investigated dental hygiene students. Both studies applied descriptive statistics and Pearson correlation analyses to identify significant prediction between empathy and burnout. The statistical significance was accepted at a *P*-value < 0.05.

The second question in the review addresses either these studies can establish dependant relationship between level of empathy and burnout. In study A students reported variables of personal (mean number = 57.9513) and work-related burnout (mean number = 57.9513); perspective taking (mean number = 2.1606) and empathetic concern (mean number = 2.2105). The level of burnout in this study was reported at a higher level with cut-off points of 50^29^. However, the overall level of burnout had negative correlations between perspective taking and empathetic concern, although not statistically significant. The statistically significant correlation was found in study B between empathy (mean number = 19.18) and emotional exhaustion (mean number = 25.51). Emotional exhaustion in this study was represented at a moderate level (range 17–26)^30^. Therefore, it can be concluded with the caution that level of burnout depends on the level of empathetic responses in dental cohort students. This finding is also consistent with the previous studies involving medical students and physicians, where empathy was inversely correlated with emotional exhaustion^14,31^.

With the respect to the third question if low level of empathy can predict a burnout, the study A reported an inverse relationship between work-related and patient-related burnout and perspective- taking, fantasy and empathetic concern but not statistically significant. It was also found that burnout was directly associated with level of personal distress, meaning that a high level of burnout implies a high level of personal distress and correlation was statistically significant. This is consistent with past literature that reported higher empathic concern (*P* < 0.05) and perspective taking (*P* < 0.001) predict lower burnout in physicians^31^; therefore, the higher the empathetic level the lower is the burnout level in healthcare professionals^32^.

This statistically significant correlation was supported by the findings in the study B where a moderate negative correlation between empathy and emotional exhaustion was reported. Therefore, the assumption can be made that lower empathy levels can predict higher levels of burnout in the dental cohort students.

## Limitations

There are a number of limitations that need to be considered when interpreting this review. The number of suitable studies was limited due to either assessing only empathy or burnout elements. The lack of studies that report on the dental student’s cohort is acknowledged also. Both studies reported female participants only, and both studies vary based on the type of course, dental science and dental hygiene respectively. There could be an account of a difference in the curriculum structure between the two disciplines. Both studies did not provide a power calculation to justify their sample size.

Another limitation of the studies is that they both used different measuring scales for empathy and burnout. However, on one side scales that assess the burnout phenomenon (CBI and MBI) are validated scales and used frequently, on the other side IRI assesses both elements of empathy: cognitive and emotional, while EQSAC does not specify what type of empathy is assessed. Besides both empathy scales are aimed at assessing empathy in general public opposing to the Jefferson Scale of Empathy that has specific scales for assessment varies healthcare disciplines. Therefore, it was difficult to compare paper’s findings with different measurement scales.

A further limitation to this review is that the measuring scales are self-reported and might be susceptible to a biased response from the students. For example, the students may complete the responses in a way to please themselves or researchers. Therefore, it is important to consider this limitation, especially when the number of participants is small. Additionally, the data extraction for this systematic review was carried out by a single researcher given that this study was undertaken as part of a master’s degree project.

## Recommendations

1. More research is needed to address dearth of studies on the association between empathy and burnout in the dental undergraduate cohort.
2. To investigate relationship of empathy and burnout with a bigger sample of the dental undergraduate cohort.
3. To develop educational interventions in dental undergraduate curriculum in order to enhance cognitive empathy when training dental professionals.

## Conclusion

Burnout is a recognised occupational phenomenon that influences clinicians’ personal and professional relationships. The purpose of this review was to investigate if there is a relationship between empathy and burnout in dental undergraduate students. Based on this review it is plausible to assume that there is a dependant relationship between level of empathy and level of burnout in the dental undergraduate students. Although, due to differences in measuring scales of empathy and burnout and a small number of participants in both studies further research is required to confirm this assumption.

## Supplementary information

PRISMA Checklist

## Data Availability

Data is derived from public domain resources.
